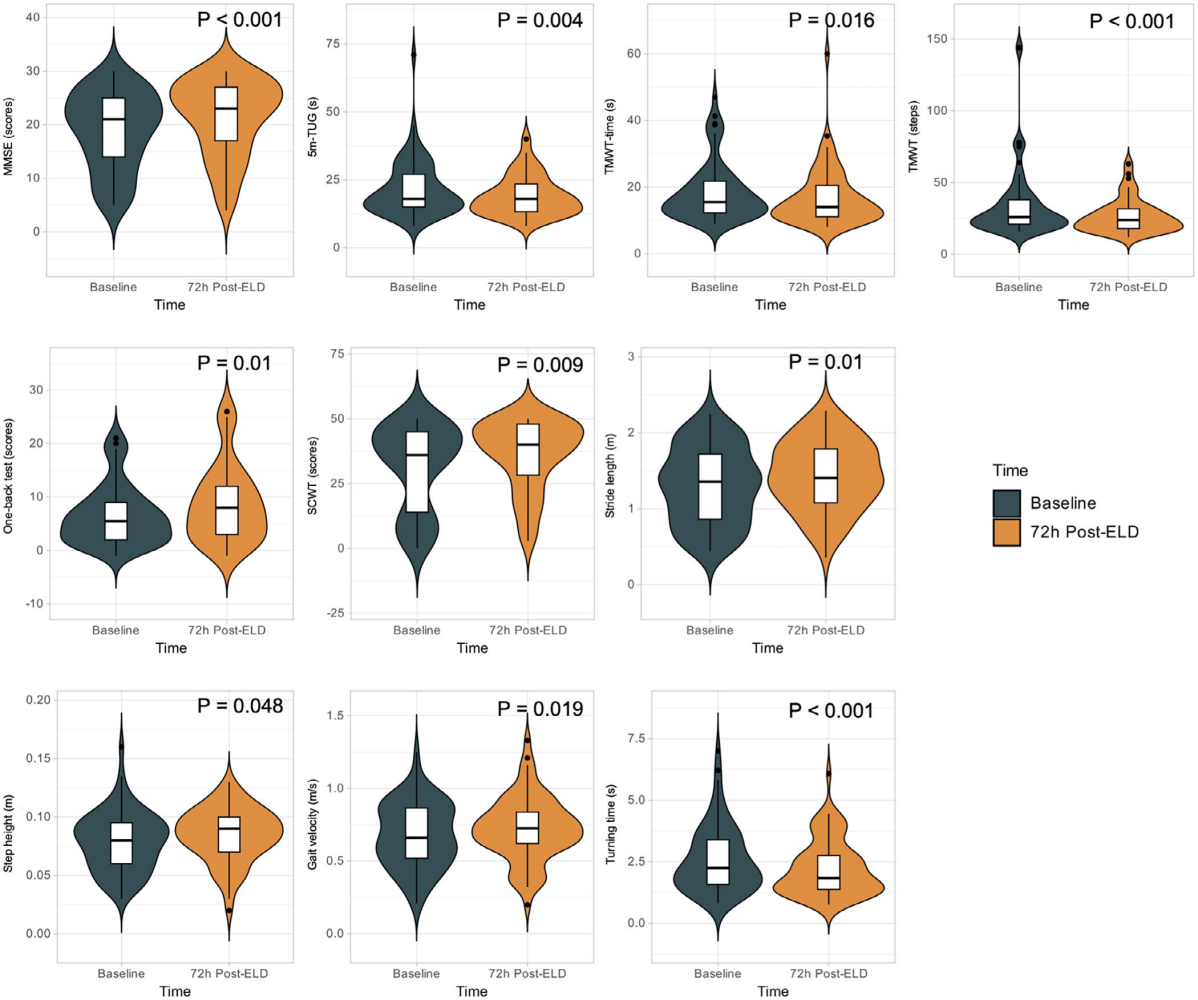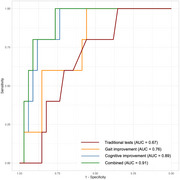# Predictive value of digital neuropsychological and gait assessments on symptomatic improvement after shunt surgery in patients with idiopathic normal pressure hydrocephalus

**DOI:** 10.1002/alz70857_105381

**Published:** 2025-12-25

**Authors:** Hanlin Cai, Keru Huang, Zilong Hao, Na Hu, Hui Gao, Feng Yang, Shiyu Feng, Linyuan Qin, Ruihan Wang, Xiyue Yang, Shan Wang, Qian Liao, Yi Liu, Dong Zhou, Liangxue Zhou, Jiaojiang He, Qin Chen

**Affiliations:** ^1^ West China Hospital of Sichuan University, Chengdu, Sichuan, China

## Abstract

**Background:**

Idiopathic normal pressure hydrocephalus (iNPH) is a treatable disease by cerebrospinal fluid (CSF) shunt. The CSF drainage test is an important tool for predicting shunt outcome in patients with iNPH, while traditional evaluation methods including mini‐mental state examination, ten‐meter walking test, and timed‐up and go test for CSF drainage tests have relatively low sensitivities. This study aimed to investigate the predictive value of digital neuropsychological and gait assessments on shunt outcomes in patients with iNPH.

**Method:**

A total of 70 patients with probable iNPH were enrolled from an ongoing prospective cohort study in the West China Hospital of Sichuan University from August 2021 to November 2023. Patients with possible iNPH underwent traditional cognitive and gait assessments as well as digital neuropsychological and gait tests at baseline and 72 hours after ELD. Patients who underwent shunt surgery were followed up at 3 months postoperatively. Outcomes were measured using the iNPH grading scale. Multivariate logistic regression models and receiver operating characteristic (ROC) analysis were used to assess the predictive value of these tests in predicting post‐shunt cognitive or gait improvement.

**Result:**

For patients with probable iNPH, the performance of the mini‐mental state examination (*p* <0.001), one‐back test (*p* = 0.01), Stroop color‐word test (*p* = 0.009), and gait analysis exhibited significant improvement 72 hours after external lumbar drainage (ELD). Thirty‐nine patients received a lumboperitoneal shunt, with 34 of them (87.2%) exhibiting cognitive or gait improvement post‐shunt. A higher average improvement rate in digital neuropsychological tests and gait performance is associated with a greater likelihood of post‐shunt response (multivariate OR = 1.06, *p* = 0.021). ROC analysis revealed that using a 10.5% average improvement rate in digital cognitive and motor assessments as the cut‐off provided the best predictive efficacy (area under ROC=0.91), which was superior to traditional tests (AUC=0.91 vs. 0.67, *p* = 0.044).

**Conclusion:**

Our study demonstrates that digital neuropsychological and gait assessments are objective evaluation methods for assessing patients with iNPH during ELD and could improve the predictive value of shunt outcomes compared to traditional tests.